# Synthesis, crystal structure and Hirshfeld surface analysis of 3-ethyl-2-(methyl­sulfan­yl)-5,5-diphenyl-3*H*-imidazol-4(5*H*)-one (Thio­phenytoin analogue)

**DOI:** 10.1107/S205698902500698X

**Published:** 2025-08-05

**Authors:** Abderrazzak El Moutaouakil Ala Allah, Chiara Massera, Walid Guerrab, Abdulsalam Alsubari, Joel T. Mague, Youssef Ramli

**Affiliations:** a Laboratory of Medicinal Chemistry Drug Sciences Research Center Faculty of Medicine and Pharmacy Mohammed V University in Rabat Morocco; b Dipartimento di Scienze Chimiche, della Vita e della Sostenibilità Ambientale, Università di Parma, Parco Area delle Scienze 17/A 43124 Parma, Italy; c Laboratory of Medicinal Chemistry, Faculty of Clinical Pharmacy, 21September University, Yemen; d Department of Chemistry, Tulane University, New Orleans, LA 70118, USA; Katholieke Universiteit Leuven, Belgium

**Keywords:** crystal structure, thio­phenytoin, di­hydro­imidazolone, methyl­sulfan­yl, Hirshfeld surface

## Abstract

In the title mol­ecule, 3-ethyl-2-(methyl­sulfan­yl)-5,5-diphenyl-3*H*-imidazol-4(5*H*)-one, C_18_H_18_N_2_OS, the two substituent phenyl rings are inclined of 59.50 (7) and 83.53 (8)° with respect to the plane of the five-membered ring. The *S*-methyl group lies in this plane while the ethyl group is nearly perpendicular to it. In the crystal, weak C—H⋯O hydrogen bonds form inversion dimers, which pack *via* conventional van der Waals contacts. A Hirshfeld surface analysis was performed, showing the predominance of H⋯H and C⋯H/H⋯C contacts.

## Abstract 2

The mol­ecular and crystal structures of 3-ethyl-2-(methyl­sulfan­yl)-5,5-diphenyl-3*H*-imidazol-4(5*H*)-one were determined and compared with the structures of similar mol­ecules obtained from the CSD. Inter­molecular inter­actions were further examined through a Hirshfeld surface analysis.

## 1. Chemical context

Hydantoin (imidazolidine-2,4-dione) represents a highly valuable and widely utilized heterocyclic scaffold in medicinal chemistry, as demonstrated by its presence in several clinically approved drugs, including phenytoin, nitro­furan­toin, and enzalutamide (El Moutaouakil Ala Allah, Guerrab *et al.*, 2024 ▸). The hydantoin scaffold exhibits a wide range of pharmacological and biological properties, including anti­bacterial (Allah *et al.*, 2024 ▸), anti­epileptic (El Moutaouakil Ala Allah, Guerrab *et al.*, 2024 ▸), anti­diabetic (Guerrab *et al.*, 2025 ▸; El Moutaouakil Ala Allah *et al.*, 2025 ▸), anti­cancer (Shankaraiah *et al.*, 2014 ▸), and anti-inflammatory (Asim Kaplancikli *et al.*, 2012 ▸) activities. Furthermore, hydantoin derivatives are well known for their broad activity in corrosion prevention (AlObaid *et al.*, 2024 ▸; Ait Mansour *et al.*, 2025 ▸), and several of them have demonstrated high corrosion inhibition efficiency (Ettahiri *et al.*, 2025 ▸; El Kaouahi *et al.*, 2025 ▸).
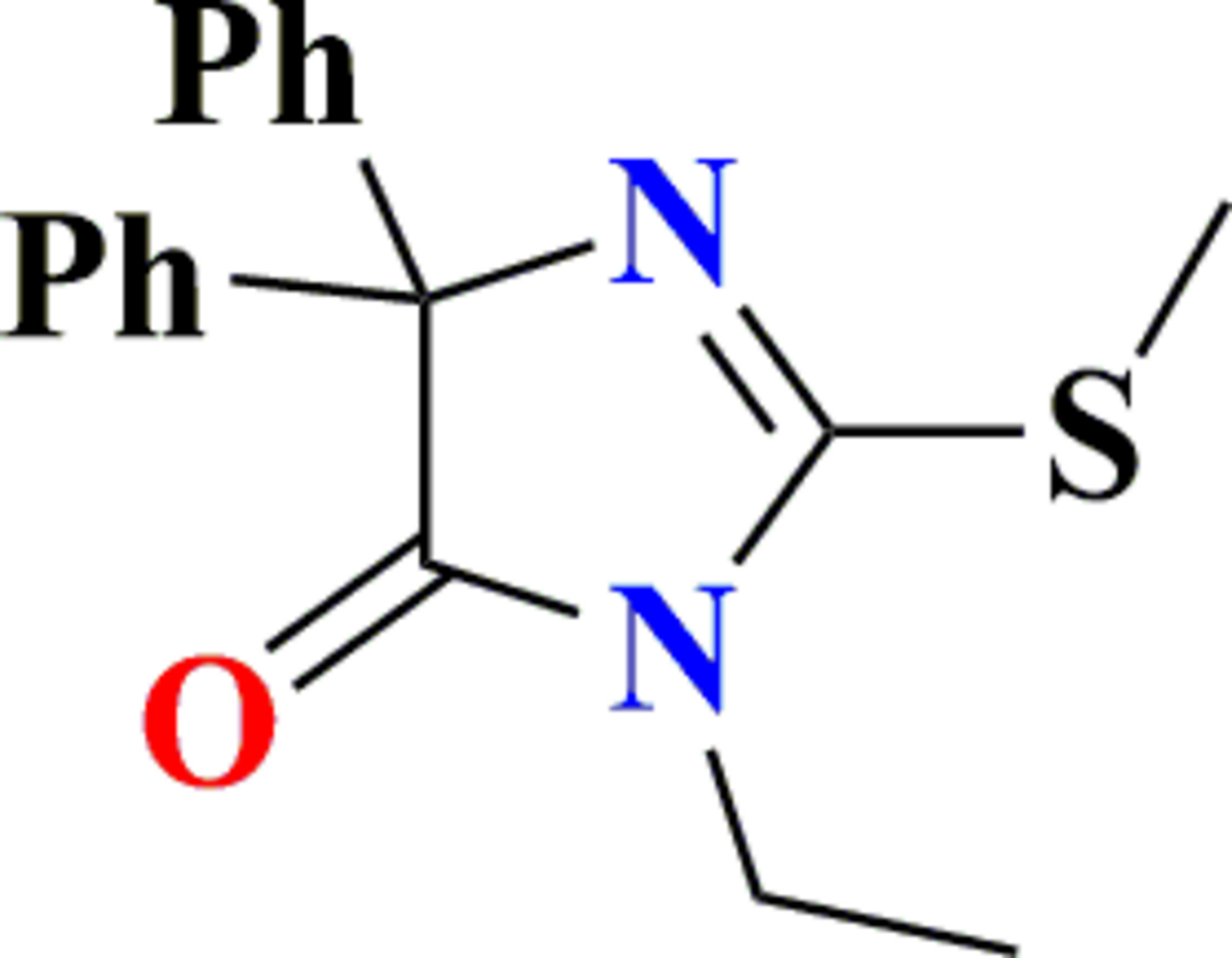


As part of our ongoing research on heterocyclic scaffolds (El Moutaouakil Ala Allah, Kariuki *et al.* 2024 ▸, El Moutaouakil Ala Allah *et al.*, 2025 ▸; Guerrab *et al.*, 2022 ▸), we report the synthesis of 3-ethyl-2-(methyl­sulfan­yl)-5,5-diphenyl-3*H*-imidazol-4(5*H*)-one, **3**, *via* an *N*-alkyl­ation reaction of 2-(methyl­sulfan­yl)-5,5-diphenyl-3*H*-imidazol-4(5*H*)-one.

## 2. Structural commentary

**Figure 1:**
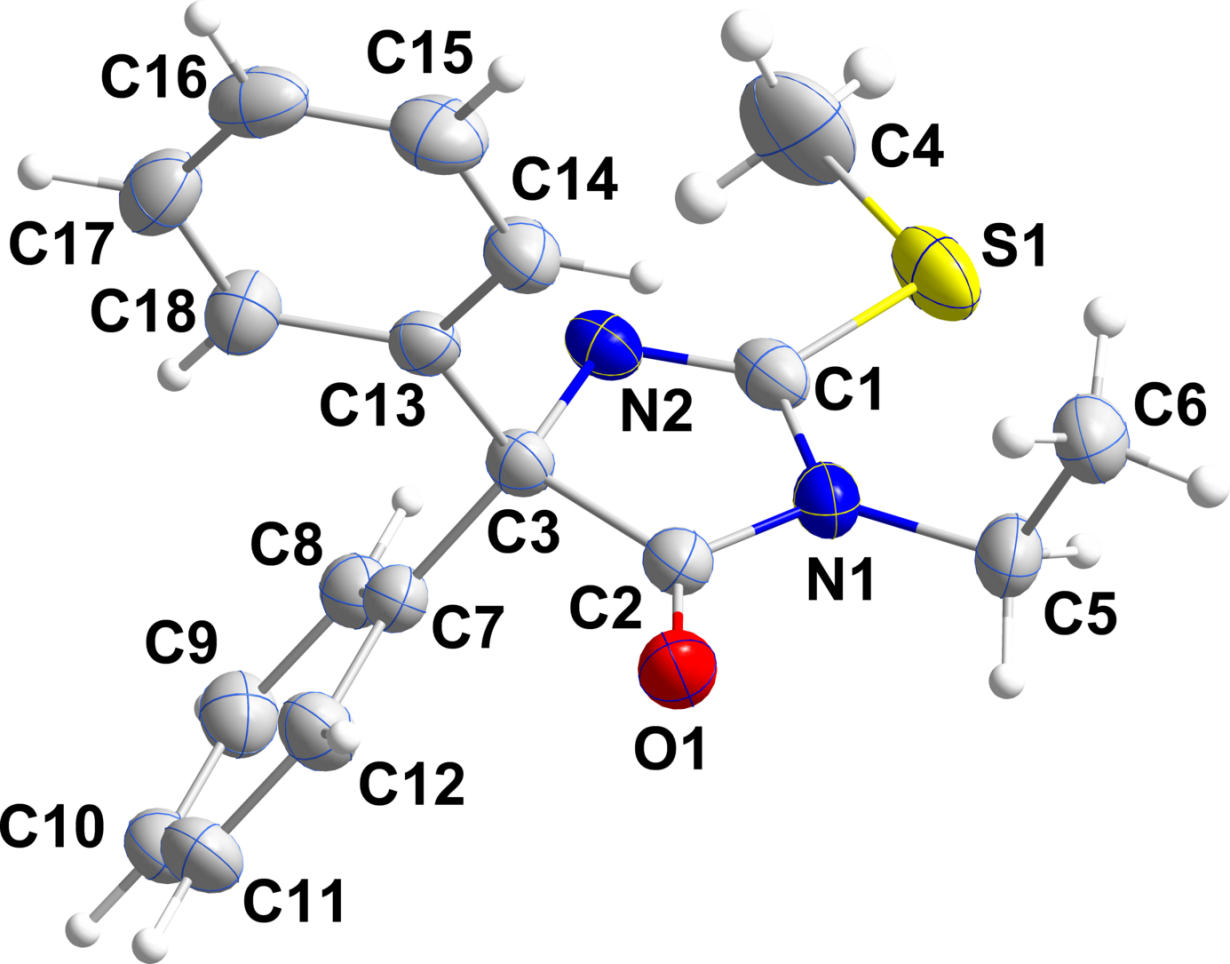
Perspective view of the title mol­ecule with labeling scheme and 50% probability ellipsoids.

The title mol­ecule crystallizes in the monoclinic space group *P*2_1_/*n* (Fig. 1 ▸). The dihedral angles between the mean plane of the five-membered ring and the planes of the C7–C12 and the C13–C18 phenyl rings are 59.50 (7) and 83.53 (8)°, respectively, which is one of the larger differences in the dihedral angles found in related mol­ecules (*vide infra*). The five-membered ring is planar to within 0.007 (1) Å (r.m.s. deviation of the fitted atoms = 0.001 Å) and the C4—S1 group lies within its plane as the C4—S1—C1—N1 torsion angle is −179.47 (13)°. In contrast, the ethyl group is nearly perpendicular to the aforementioned plane as the C1—N1—C5—C6 torsion angle is 87.81 (19)°.

## 3. Supra­molecular features

**Figure 2:**
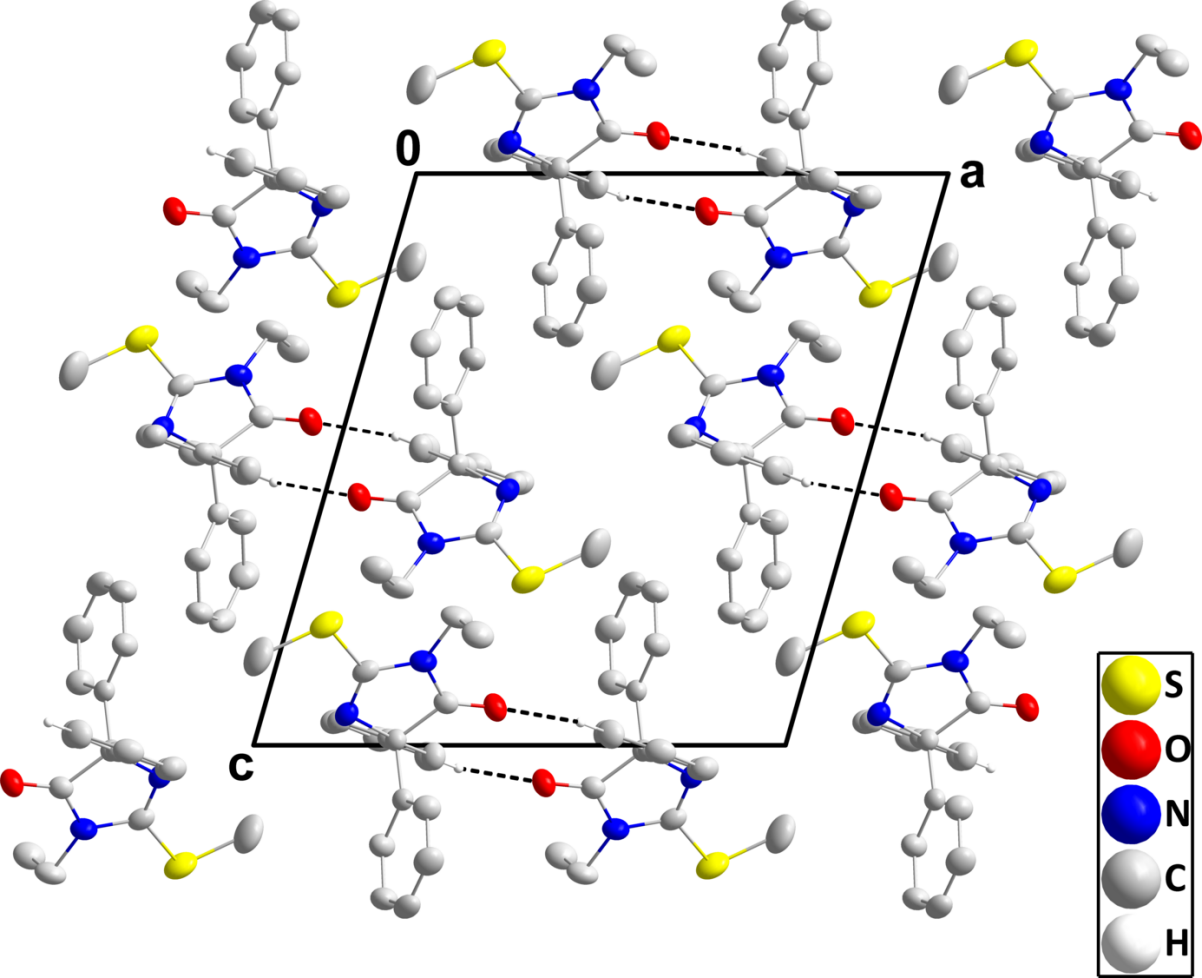
Packing viewed along the *b*-axis direction with C—H⋯O hydrogen bonds depicted by dashed lines and hydrogen atoms not participating in these inter­actions omitted for clarity.

In the crystal, weak C11—H11⋯O1^i^ hydrogen bonds (Table 1 ▸) form centrosymmetric dimers, which, in turn, pack *via* conventional van der Waals contacts (Fig. 2 ▸).

## 4. Database survey

**Figure 3:**
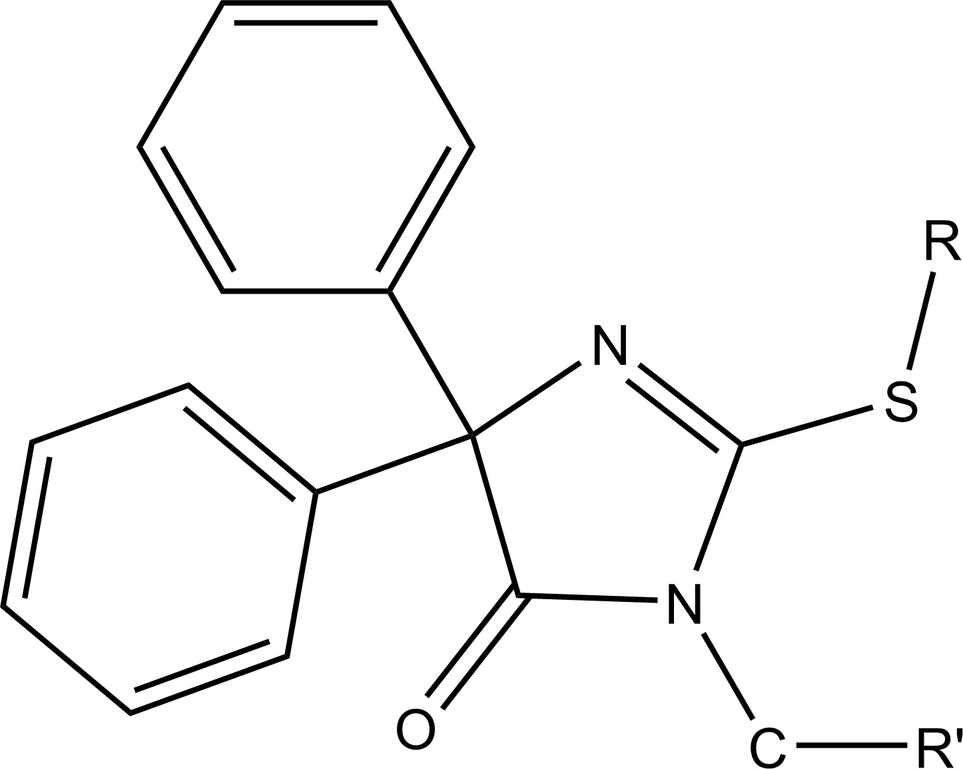
The fragment used in the Cambridge Structural Database search.

A search of the Cambridge Structural Database (CSD, updated to May 2025 (Groom *et al.*, 2016 ▸) with the fragment shown in Fig. 3 ▸, where *R* = *R*′ = no substituent, yielded nine hits. Included mol­ecules have *R*,*R*′ = –CH_2_CH_2_– (DIYRAE; Karolak-Wojciechowska *et al.*, 1985 ▸), –CH_2_CH(COOEt)– (FURFED; Karolak-Wojciechowska & Kieć-Kononowicz, 1987 ▸), –CH_2_CH_2_CH_2_– (IMTHZN; Kieć-Kononowicz *et al.*, 1981 ▸ and IMTHZN01; Guerrab *et al.*, 2019 ▸), and –CH_2_CH_2_OCH_2_CH_2_OCH_2_CH_2_OCH_2_CH_2_– (LIGWOR; Guerrab *et al.*, 2023 ▸) as well as *R* = *R*′ = benzyl (RAHGUF; Akrad *et al.*, 2017 ▸), *R* = *R*′ = *n*-propyl (RIJZIW; Akrad *et al.*, 2018 ▸), *R* = *R*′ = methyl (YEYYUA; El Moutaouakil Ala Allah *et al.*, 2023 ▸) and *R* = *R*′ = ethyl (HOPQAI; El Moutaouakil Ala Allah, Guerrab *et al.*, 2024 ▸). The dihedral angles between the planes of the two phenyl rings attached directly to the 4,5-di­hydro-1*H*-imidazol-5-one ring vary over the range 47.89° to 89.59° due to the differing packings resulting from the varied sizes and shapes of the *R* and *R*′ substituents. In most instances, the two angles differ by *ca*. 15° but in LIGWOR they are nearly equal, being 62.10 (11) and 61.35 (11)°. As in the title mol­ecule, the packing in RAHGOF and HOPQAI involves the formation of centrosymmetric dimers through weak C—H⋯O hydrogen bonds, with dimers associated through van der Waals inter­actions. In all of the other compounds, except for IMTHZN and IMTHZN01, chains of mol­ecules parallel to crystallographic *a* axis are generated by weak C—H⋯O hydrogen bonds. In the exceptions, the chain is formed by weak C—H⋯N hydrogen bonds and the chains are linked by weak C—H⋯O hydrogen bonds and C—H⋯π(ring) inter­actions.

## 5. Hirshfeld surface analysis

**Figure 4:**
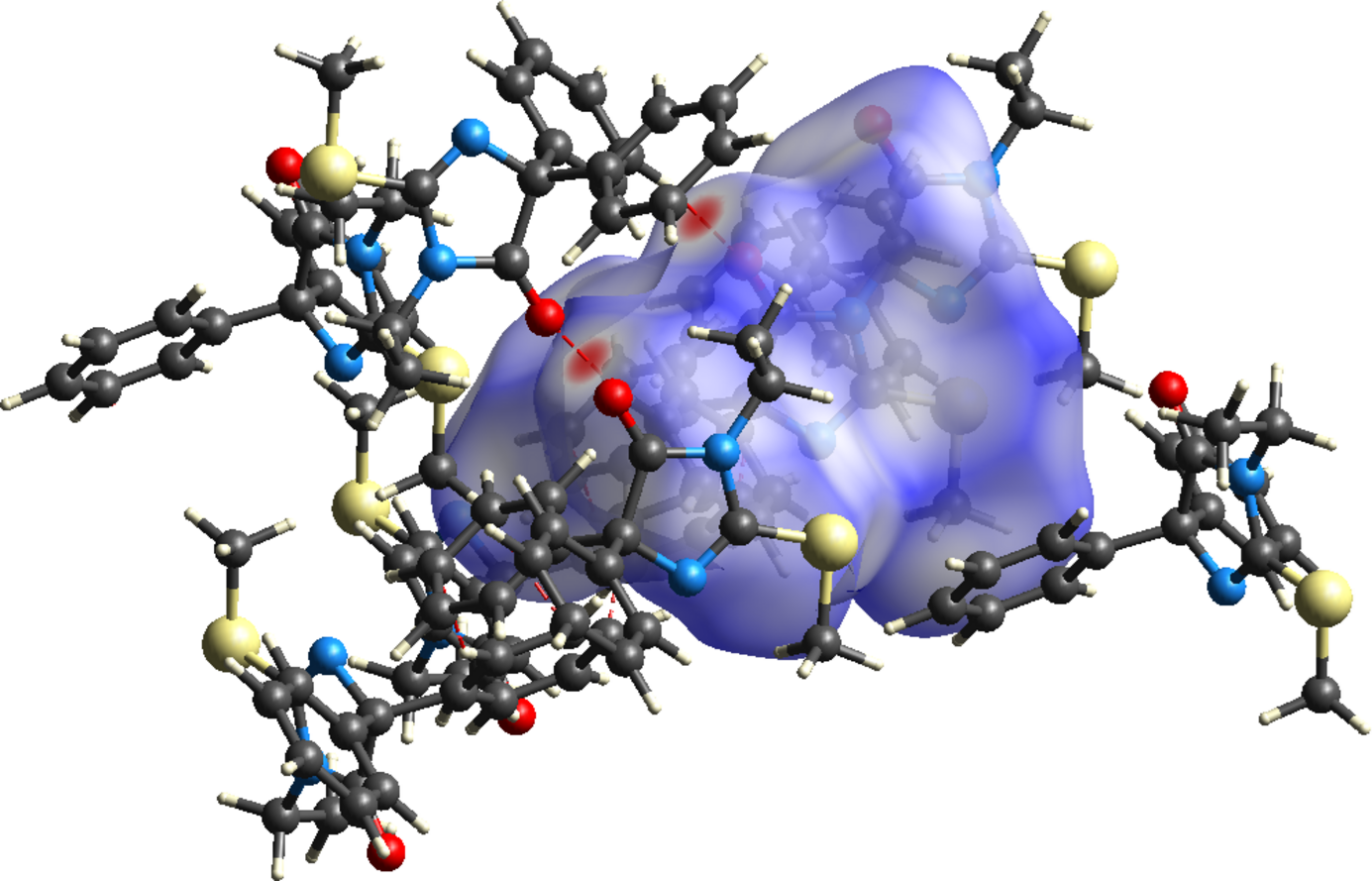
The Hirshfeld *d*_norm_ surface of the title compound with several neighboring mol­ecules. The C—H⋯O hydrogen bonds are depicted by red dashed lines.

**Figure 5:**
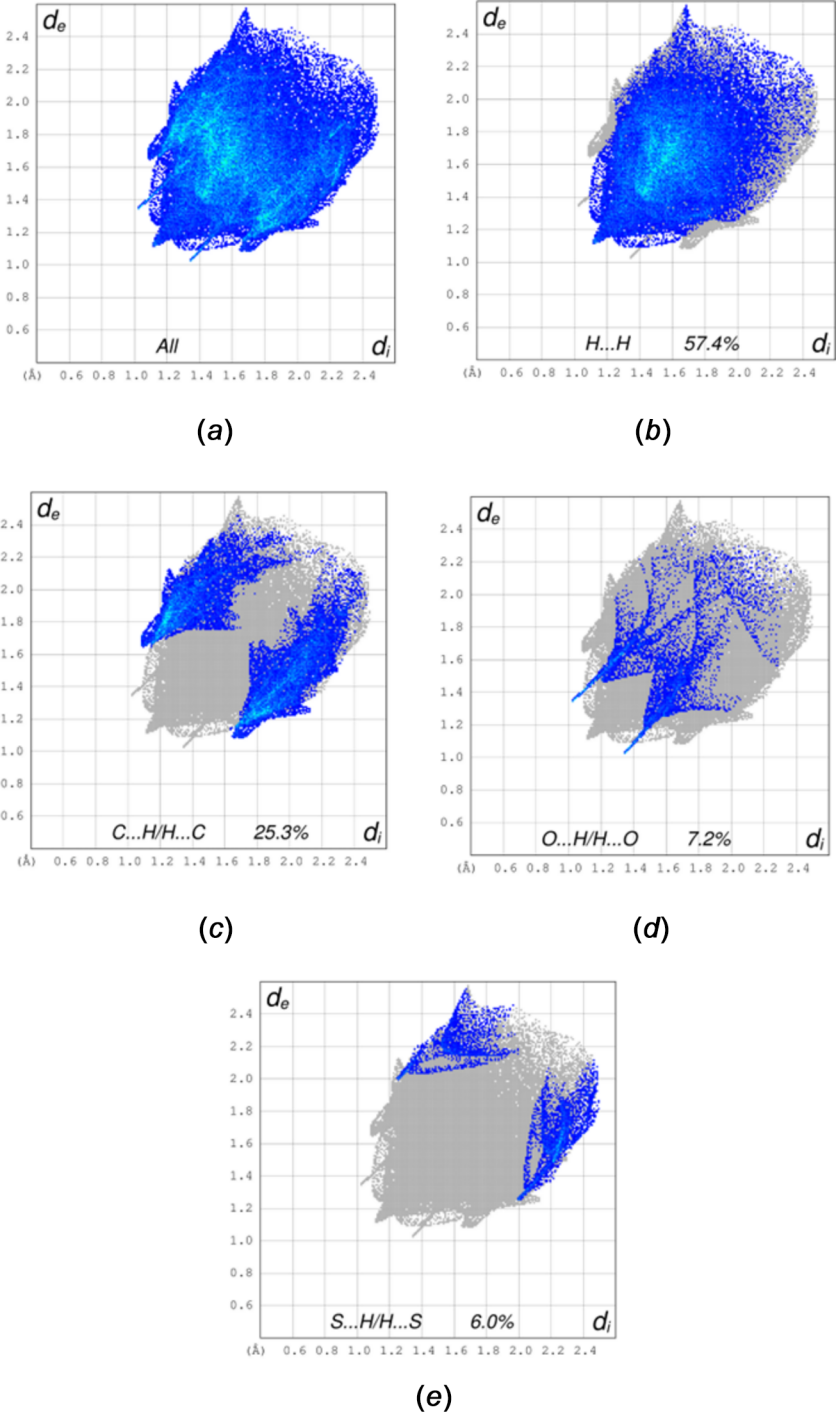
The 2-D fingerprint plots showing all inter­molecular contacts (*a*) and those delineated into H⋯H (*b*), C⋯H/H⋯C (*c*), O⋯H/H⋯O (*d*) and S⋯H/H⋯S (***e***) contacts.

The *d*_norm_ surface and 2-D fingerprint plots for the title mol­ecule were calculated with *CrystalExplorer* (Spackman *et al.*, 2021 ▸) and full descriptions of the methods and inter­pretations of the results have been published by Tan *et al.* (2019 ▸). The *d*_norm_ surface together with several neighboring mol­ecules is shown in Fig. 4 ▸, in which the C—H⋯O hydrogen bonds are shown as red dashed lines passing through the dark red spots on the surface (indicating contacts shorter than the sum of the van der Waals radii). Fig. 5 ▸ shows the 2-D fingerprint plots for all inter­molecular inter­actions (*a*) and those delineated into contacts between specific atom types (*b*)–(*e*). The H⋯H contacts (*b*) account for 57.4% of the total, which is expected as the periphery of the mol­ecule consists largely of hydrogen atoms. Most are bound to phenyl and methyl groups, which are directed outwards from the center of gravity and so will be the first to contact neighboring mol­ecules. It is perhaps surprising that the C⋯H/H⋯C contacts (*c*), which contribute 25.3% of the total, are more prevalent than the O⋯H/H⋯O contacts (*d*, 7.2%), despite the latter being the only inter­actions in the packing that can be regarded as directional (*vide supra*). However, the geometrical analysis carried out with *PLATON* (Spek, 2020 ▸) shows a contact of 2.94 Å between H5*B* and the centroid *Cg* of the C7–C12 phenyl ring at −*x* + 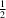, *y* + 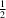, −*z* + 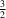. Nevertheless, the C5—H5*B*⋯C*g*1 angle of 127° is quite small for the contact to be considered a definite C—H⋯π(ring) inter­action. Several other C⋯H distances approximately equal to the sum of the two van der Waals radii are listed, which can account for this high percentage contribution. As noted, the O⋯H/H⋯O contacts come primarily from the weak C—H⋯O hydrogen bonds described in Table 1 ▸. Although the S⋯H/H⋯S inter­actions contribute almost as much, there does not appear to be any obvious C—H⋯S hydrogen bond.

## 6. Synthesis and crystallization

**Figure 6:**
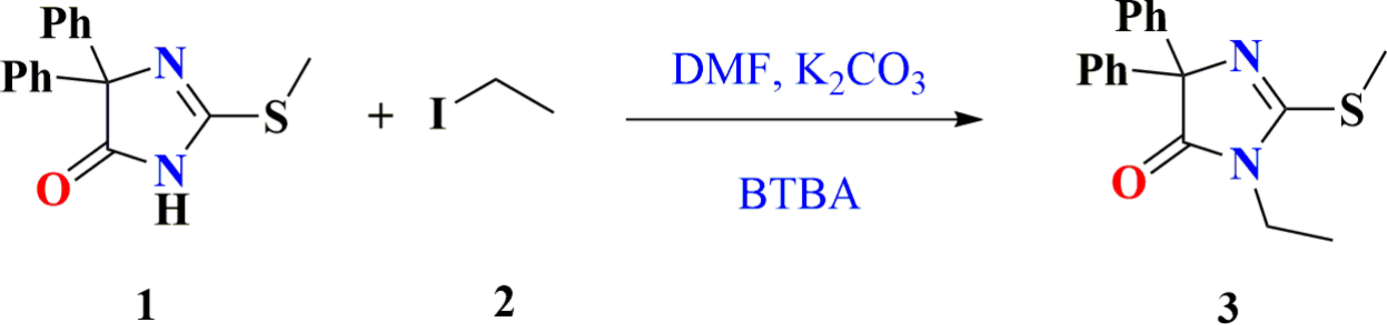
Synthesis of the title compound.

The title compound was obtained according to the reaction scheme shown in Fig. 6 ▸. To a solution of 2-(methyl­sulfan­yl)-5,5-diphenyl-3*H*-imidazol-4(5*H*)-one (**1**) (0.5 g, 1.7 mmol) in DMF (10 mL), iodo­ethane (**2**) (2.10 mmol) was added in the presence of K_2_CO_3_ (1.8 mmol) and a catalytic amount of BTBA (10%). The reaction mixture was stirred at room temperature for 3 h (El Moutaouakil Ala Allah *et al.*, 2023 ▸; Guerrab *et al.*, 2023 ▸; El Moutaouakil Ala Allah, Kariuki, Alsubari *et al.*, 2024 ▸). After filtration of the inorganic salts, the solvent was evaporated under reduced pressure, and the crude residue was purified by recrystallization from ethanol, affording 3-ethyl-2-(methyl­sulfan­yl)-5,5-diphenyl-3*H*-imid­azol-4(5*H*)-one (**3**) in 96% yield, m.p. = 394–396 K. **FT-IR** (ATR, ν, cm^−1^): 3061 (C—H Ar), 2980 (–CH_3_), 2854 (C—H Aliphatic), 1726 (C=O); **^1^H NMR** (500 MHz, CDCl_3_): δ ppm 1.24 (*t*, 3H, N—CH_2_—CH_3_), 2.70 (*s*, 3H, S—CH_3_), 3.56 (*q*, 2H, N—CH_2_—CH_3_), 7.25–7.56 (*m*, 10H, Ar H); **^13^C NMR** (125 MHz, CDCl_3_); 12.85 (N—CH_2_—CH_3_), 14.29 (S—CH_3_), 35.96 (N—CH_2_—CH_3_), 78.47 (C—2Ph); 127.24, 127.73, 128.48, 140.67 (C—Ar), 161.67 (C=N), 180,67 (C=O); **HRMS** (ESI-MS) (*m*/*z*) calculated for C_18_H_18_N_2_OS 311.1140; found 311.12036.

## 7. Refinement

Crystal data, data collection and structure refinement details are summarized in Table 2 ▸. The carbon-bound H atoms were placed in calculated positions and refined isotropically using the riding model, with C—H distances ranging from 0.95 to 0.99 Å and *U*_iso_(H) set to 1.2–1.5 *U*_eq_(C).

## Supplementary Material

Crystal structure: contains datablock(s) I. DOI: 10.1107/S205698902500698X/vm2316sup1.cif

Structure factors: contains datablock(s) I. DOI: 10.1107/S205698902500698X/vm2316Isup2.hkl

Supporting information file. DOI: 10.1107/S205698902500698X/vm2316Isup3.cml

CCDC reference: 2478215

Additional supporting information:  crystallographic information; 3D view; checkCIF report

## Figures and Tables

**Table 1:** Hydrogen-bond geometry (Å, °)

*D*—H⋯*A*	*D*—H	H⋯*A*	*D*⋯*A*	*D*—H⋯*A*
C11—H11⋯O1^i^	0.95	2.49	3.434 (2)	171

Symmetry code: (i) 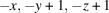.

**Table 2:** Experimental details

Crystal data	
Chemical formula	C_18_H_18_N_2_OS
*M*_r_	310.40
Crystal system, space group	Monoclinic, *P*2_1_/*n*
Temperature (K)	200
*a*, *b*, *c* (Å)	12.6759 (2), 9.2109 (2), 14.1568 (3)
β (°)	105.915 (1)
*V* (Å^3^)	1589.54 (5)
*Z*	4
Radiation type	Cu *K*α
μ (mm^−1^)	1.82
Crystal size (mm)	0.19 × 0.15 × 0.14

Data collection	
Diffractometer	Bruker D8 Venture PhotonII
Absorption correction	Multi-scan (*SADABS*; Krause *et al.*, 2015 ▸)
*T*_min_, *T*_max_	0.58, 0.75
No. of measured, independent and observed [*I* > 2σ(*I*)] reflections	15894, 3237, 2875
*R*_int_	0.047
(sin θ/λ)_max_ (Å^−1^)	0.625

Refinement	
*R*[*F*^2^ > 2σ(*F*^2^)], *wR*(*F*^2^), *S*	0.038, 0.101, 1.05
No. of reflections	3237
No. of parameters	202
H-atom treatment	H-atom parameters constrained
Δρ_max_, Δρ_min_ (e Å^−3^)	0.23, −0.33

Computer programs: *APEX3* and *SAINT* (Bruker, 2016 ▸), *SHELXT2018/2* (Sheldrick, 2015*a* ▸), *SHELXL2019/2* (Sheldrick, 2015*b* ▸), *DIAMOND* (Brandenburg & Putz, 2012 ▸), *WinGX* (Farrugia, 2012 ▸), *publCIF* (Westrip, 2010 ▸) and *enCIFer* (Allen *et al.*, 2004 ▸).

## supplementary crystallographic information

### 3-Ethyl-2-(methylsulfanyl)-5,5-diphenyl-3H-imidazol-4(5H)-one Crystal data

**Table:**

C_18_H_18_N_2_OS	*F*(000) = 656
*M**_r_* = 310.40	*D*_x_ = 1.297 Mg m^−^^3^
Monoclinic, *P*2_1_/*n*	Cu *K*α radiation, λ = 1.54178 Å
*a* = 12.6759 (2) Å	Cell parameters from 438 reflections
*b* = 9.2109 (2) Å	θ = 4.2–74.6°
*c* = 14.1568 (3) Å	µ = 1.82 mm^−^^1^
β = 105.915 (1)°	*T* = 200 K
*V* = 1589.54 (5) Å^3^	Prism, colourless
*Z* = 4	0.19 × 0.15 × 0.14 mm

### 3-Ethyl-2-(methylsulfanyl)-5,5-diphenyl-3H-imidazol-4(5H)-one Data collection

**Table:**

Bruker D8 Venture PhotonII diffractometer	3237 independent reflections
Radiation source: fine-focus sealed tube	2875 reflections with *I* > 2σ(*I*)
Graphite monochromator	*R*_int_ = 0.047
phi & ω scan	θ_max_ = 74.6°, θ_min_ = 4.2°
Absorption correction: multi-scan (SADABS; Krause *et al.*, 2015)	*h* = −15→15
*T*_min_ = 0.58, *T*_max_ = 0.75	*k* = −11→10
15894 measured reflections	*l* = −17→16

### 3-Ethyl-2-(methylsulfanyl)-5,5-diphenyl-3H-imidazol-4(5H)-one Refinement

**Table:**

Refinement on *F*^2^	Secondary atom site location: difference Fourier map
Least-squares matrix: full	Hydrogen site location: inferred from neighbouring sites
*R*[*F*^2^ > 2σ(*F*^2^)] = 0.038	H-atom parameters constrained
*wR*(*F*^2^) = 0.101	*w* = 1/[σ^2^(*F*_o_^2^) + (0.0474*P*)^2^ + 0.4531*P*] where *P* = (*F*_o_^2^ + 2*F*_c_^2^)/3
*S* = 1.05	(Δ/σ)_max_ < 0.001
3237 reflections	Δρ_max_ = 0.23 e Å^−^^3^
202 parameters	Δρ_min_ = −0.33 e Å^−^^3^
0 restraints	Extinction correction: SHELXL-2019/2 (Sheldrick 2019), Fc^*^=kFc[1+0.001xFc^2^λ^3^/sin(2θ)]^-1/4^
Primary atom site location: dual	Extinction coefficient: 0.0025 (4)

### 3-Ethyl-2-(methylsulfanyl)-5,5-diphenyl-3H-imidazol-4(5H)-one Special details

**Table:**

Geometry. All esds (except the esd in the dihedral angle between two l.s. planes) are estimated using the full covariance matrix. The cell esds are taken into account individually in the estimation of esds in distances, angles and torsion angles; correlations between esds in cell parameters are only used when they are defined by crystal symmetry. An approximate (isotropic) treatment of cell esds is used for estimating esds involving l.s. planes.

### 3-Ethyl-2-(methylsulfanyl)-5,5-diphenyl-3H-imidazol-4(5H)-one Fractional atomic coordinates and isotropic or equivalent isotropic displacement parameters (Å^2^)

**Table:**

	*x*	*y*	*z*	*U*_iso_*/*U*_eq_	
S1	0.42759 (4)	1.04706 (4)	0.71077 (3)	0.05142 (16)	
O1	0.06602 (8)	0.81371 (11)	0.56610 (8)	0.0416 (3)	
N2	0.34143 (9)	0.87359 (12)	0.55450 (8)	0.0325 (3)	
N1	0.22587 (10)	0.92996 (13)	0.64679 (8)	0.0356 (3)	
C13	0.19033 (10)	0.85995 (14)	0.40490 (9)	0.0292 (3)	
C3	0.23515 (10)	0.80042 (14)	0.50922 (9)	0.0287 (3)	
C7	0.24828 (10)	0.63546 (14)	0.51131 (9)	0.0295 (3)	
C2	0.16102 (11)	0.84460 (14)	0.57434 (10)	0.0317 (3)	
C1	0.32858 (11)	0.94226 (14)	0.62915 (10)	0.0337 (3)	
C8	0.35072 (11)	0.57087 (15)	0.54165 (10)	0.0337 (3)	
H8	0.414523	0.629466	0.562886	0.040*	
C18	0.20459 (12)	0.78517 (17)	0.32453 (10)	0.0396 (3)	
H18	0.236884	0.691312	0.333256	0.047*	
C12	0.15565 (12)	0.54770 (16)	0.48046 (12)	0.0390 (3)	
H12	0.085017	0.590867	0.459211	0.047*	
C10	0.26845 (13)	0.33415 (16)	0.51073 (11)	0.0416 (3)	
H10	0.275616	0.231491	0.510549	0.050*	
C9	0.36044 (13)	0.42047 (16)	0.54111 (11)	0.0400 (3)	
H9	0.430967	0.376892	0.561814	0.048*	
C14	0.14038 (12)	0.99562 (16)	0.39013 (11)	0.0391 (3)	
H14	0.128113	1.047293	0.444221	0.047*	
C17	0.17226 (13)	0.84580 (19)	0.23138 (11)	0.0454 (4)	
H17	0.182748	0.793562	0.176805	0.054*	
C11	0.16586 (13)	0.39736 (17)	0.48057 (12)	0.0442 (4)	
H11	0.102309	0.338169	0.459910	0.053*	
C5	0.18677 (15)	0.99957 (17)	0.72403 (11)	0.0437 (4)	
H5B	0.248783	1.009702	0.783927	0.052*	
H5A	0.130796	0.936822	0.740302	0.052*	
C16	0.12491 (12)	0.98171 (19)	0.21774 (11)	0.0447 (4)	
H16	0.103796	1.023941	0.154098	0.054*	
C15	0.10842 (14)	1.05583 (18)	0.29694 (13)	0.0477 (4)	
H15	0.074882	1.148914	0.287611	0.057*	
C6	0.13762 (15)	1.14777 (17)	0.69332 (12)	0.0493 (4)	
H6A	0.079291	1.139169	0.631694	0.074*	
H6C	0.194766	1.213302	0.683826	0.074*	
H6B	0.107005	1.186788	0.744561	0.074*	
C4	0.53801 (16)	1.0279 (3)	0.65510 (19)	0.0739 (6)	
H4C	0.519234	1.078181	0.591672	0.111*	
H4B	0.550231	0.924663	0.644994	0.111*	
H4A	0.604906	1.070302	0.698209	0.111*	

### 3-Ethyl-2-(methylsulfanyl)-5,5-diphenyl-3H-imidazol-4(5H)-one Atomic displacement parameters (Å^2^)

**Table:**

	*U*^11^	*U*^22^	*U*^33^	*U*^12^	*U*^13^	*U*^23^
S1	0.0557 (3)	0.0418 (2)	0.0454 (2)	−0.01332 (17)	−0.00529 (18)	−0.00844 (16)
O1	0.0385 (5)	0.0426 (6)	0.0487 (6)	−0.0066 (4)	0.0205 (4)	−0.0076 (5)
N2	0.0308 (5)	0.0286 (6)	0.0351 (6)	−0.0050 (4)	0.0042 (4)	−0.0001 (5)
N1	0.0449 (6)	0.0318 (6)	0.0299 (6)	−0.0034 (5)	0.0098 (5)	−0.0047 (5)
C13	0.0271 (6)	0.0298 (6)	0.0304 (6)	−0.0048 (5)	0.0075 (5)	0.0000 (5)
C3	0.0279 (6)	0.0279 (6)	0.0299 (6)	−0.0031 (5)	0.0071 (5)	−0.0024 (5)
C7	0.0348 (6)	0.0286 (6)	0.0258 (6)	−0.0027 (5)	0.0096 (5)	−0.0013 (5)
C2	0.0379 (7)	0.0270 (6)	0.0308 (6)	−0.0016 (5)	0.0107 (5)	−0.0002 (5)
C1	0.0385 (7)	0.0264 (6)	0.0312 (7)	−0.0038 (5)	0.0008 (5)	0.0018 (5)
C8	0.0370 (7)	0.0328 (7)	0.0306 (6)	−0.0016 (5)	0.0080 (5)	−0.0013 (5)
C18	0.0423 (8)	0.0423 (8)	0.0350 (7)	0.0047 (6)	0.0121 (6)	−0.0012 (6)
C12	0.0361 (7)	0.0339 (7)	0.0473 (8)	−0.0045 (5)	0.0119 (6)	−0.0053 (6)
C10	0.0604 (9)	0.0275 (7)	0.0385 (7)	−0.0016 (6)	0.0161 (7)	0.0004 (6)
C9	0.0460 (8)	0.0357 (7)	0.0363 (7)	0.0063 (6)	0.0075 (6)	0.0010 (6)
C14	0.0480 (8)	0.0296 (7)	0.0393 (7)	−0.0004 (6)	0.0114 (6)	0.0000 (6)
C17	0.0473 (8)	0.0577 (10)	0.0318 (7)	−0.0036 (7)	0.0120 (6)	−0.0009 (7)
C11	0.0481 (8)	0.0347 (8)	0.0514 (9)	−0.0126 (6)	0.0166 (7)	−0.0069 (7)
C5	0.0641 (10)	0.0384 (8)	0.0314 (7)	−0.0014 (7)	0.0177 (7)	−0.0058 (6)
C16	0.0404 (8)	0.0540 (9)	0.0358 (7)	−0.0114 (7)	0.0037 (6)	0.0126 (7)
C15	0.0531 (9)	0.0372 (8)	0.0500 (9)	0.0012 (7)	0.0093 (7)	0.0116 (7)
C6	0.0661 (10)	0.0375 (8)	0.0469 (9)	−0.0004 (7)	0.0200 (8)	−0.0072 (7)
C4	0.0447 (10)	0.0732 (14)	0.0939 (16)	−0.0214 (9)	0.0023 (10)	−0.0164 (12)

### 3-Ethyl-2-(methylsulfanyl)-5,5-diphenyl-3H-imidazol-4(5H)-one Geometric parameters (Å, º)

**Table:**

S1—C1	1.7455 (14)	C10—C9	1.379 (2)
S1—C4	1.793 (2)	C10—C11	1.381 (2)
O1—C2	1.2116 (16)	C10—H10	0.9500
N2—C1	1.2797 (18)	C9—H9	0.9500
N2—C3	1.4855 (16)	C14—C15	1.385 (2)
N1—C2	1.3723 (17)	C14—H14	0.9500
N1—C1	1.3960 (19)	C17—C16	1.379 (2)
N1—C5	1.4663 (18)	C17—H17	0.9500
C13—C18	1.3837 (19)	C11—H11	0.9500
C13—C14	1.3906 (19)	C5—C6	1.514 (2)
C13—C3	1.5311 (17)	C5—H5B	0.9900
C3—C7	1.5279 (18)	C5—H5A	0.9900
C3—C2	1.5405 (18)	C16—C15	1.377 (2)
C7—C8	1.3851 (19)	C16—H16	0.9500
C7—C12	1.3934 (19)	C15—H15	0.9500
C8—C9	1.391 (2)	C6—H6A	0.9800
C8—H8	0.9500	C6—H6C	0.9800
C18—C17	1.386 (2)	C6—H6B	0.9800
C18—H18	0.9500	C4—H4C	0.9800
C12—C11	1.391 (2)	C4—H4B	0.9800
C12—H12	0.9500	C4—H4A	0.9800
			
C1—S1—C4	99.25 (8)	C10—C9—H9	119.8
C1—N2—C3	105.96 (11)	C8—C9—H9	119.8
C2—N1—C1	108.04 (11)	C15—C14—C13	120.35 (14)
C2—N1—C5	123.52 (13)	C15—C14—H14	119.8
C1—N1—C5	128.35 (12)	C13—C14—H14	119.8
C18—C13—C14	118.71 (13)	C16—C17—C18	120.18 (14)
C18—C13—C3	121.15 (12)	C16—C17—H17	119.9
C14—C13—C3	120.01 (12)	C18—C17—H17	119.9
N2—C3—C7	111.21 (10)	C10—C11—C12	119.97 (14)
N2—C3—C13	107.85 (10)	C10—C11—H11	120.0
C7—C3—C13	112.61 (10)	C12—C11—H11	120.0
N2—C3—C2	104.58 (10)	N1—C5—C6	112.13 (12)
C7—C3—C2	109.41 (10)	N1—C5—H5B	109.2
C13—C3—C2	110.90 (10)	C6—C5—H5B	109.2
C8—C7—C12	119.06 (13)	N1—C5—H5A	109.2
C8—C7—C3	121.40 (11)	C6—C5—H5A	109.2
C12—C7—C3	119.53 (12)	H5B—C5—H5A	107.9
O1—C2—N1	125.61 (12)	C15—C16—C17	119.58 (14)
O1—C2—C3	129.32 (12)	C15—C16—H16	120.2
N1—C2—C3	105.07 (11)	C17—C16—H16	120.2
N2—C1—N1	116.34 (12)	C16—C15—C14	120.46 (15)
N2—C1—S1	126.07 (11)	C16—C15—H15	119.8
N1—C1—S1	117.58 (10)	C14—C15—H15	119.8
C7—C8—C9	120.20 (13)	C5—C6—H6A	109.5
C7—C8—H8	119.9	C5—C6—H6C	109.5
C9—C8—H8	119.9	H6A—C6—H6C	109.5
C13—C18—C17	120.69 (14)	C5—C6—H6B	109.5
C13—C18—H18	119.7	H6A—C6—H6B	109.5
C17—C18—H18	119.7	H6C—C6—H6B	109.5
C11—C12—C7	120.47 (14)	S1—C4—H4C	109.5
C11—C12—H12	119.8	S1—C4—H4B	109.5
C7—C12—H12	119.8	H4C—C4—H4B	109.5
C9—C10—C11	119.82 (14)	S1—C4—H4A	109.5
C9—C10—H10	120.1	H4C—C4—H4A	109.5
C11—C10—H10	120.1	H4B—C4—H4A	109.5
C10—C9—C8	120.48 (14)		
			
C1—N2—C3—C7	−117.50 (12)	C3—N2—C1—S1	179.54 (10)
C1—N2—C3—C13	118.57 (12)	C2—N1—C1—N2	−1.10 (16)
C1—N2—C3—C2	0.48 (13)	C5—N1—C1—N2	−177.81 (13)
C18—C13—C3—N2	98.09 (14)	C2—N1—C1—S1	179.61 (9)
C14—C13—C3—N2	−77.78 (14)	C5—N1—C1—S1	2.90 (19)
C18—C13—C3—C7	−24.99 (17)	C4—S1—C1—N2	1.33 (15)
C14—C13—C3—C7	159.15 (12)	C4—S1—C1—N1	−179.47 (13)
C18—C13—C3—C2	−147.96 (12)	C12—C7—C8—C9	0.0 (2)
C14—C13—C3—C2	36.17 (16)	C3—C7—C8—C9	−178.82 (12)
N2—C3—C7—C8	−7.14 (16)	C14—C13—C18—C17	1.6 (2)
C13—C3—C7—C8	114.04 (13)	C3—C13—C18—C17	−174.32 (13)
C2—C3—C7—C8	−122.16 (13)	C8—C7—C12—C11	0.3 (2)
N2—C3—C7—C12	174.01 (12)	C3—C7—C12—C11	179.17 (13)
C13—C3—C7—C12	−64.81 (15)	C11—C10—C9—C8	0.0 (2)
C2—C3—C7—C12	58.99 (15)	C7—C8—C9—C10	−0.2 (2)
C1—N1—C2—O1	−178.57 (13)	C18—C13—C14—C15	−1.7 (2)
C5—N1—C2—O1	−1.7 (2)	C3—C13—C14—C15	174.24 (13)
C1—N1—C2—C3	1.28 (14)	C13—C18—C17—C16	−0.3 (2)
C5—N1—C2—C3	178.18 (12)	C9—C10—C11—C12	0.3 (2)
N2—C3—C2—O1	178.75 (14)	C7—C12—C11—C10	−0.5 (2)
C7—C3—C2—O1	−62.04 (18)	C2—N1—C5—C6	−88.44 (17)
C13—C3—C2—O1	62.76 (18)	C1—N1—C5—C6	87.81 (19)
N2—C3—C2—N1	−1.09 (13)	C18—C17—C16—C15	−1.0 (2)
C7—C3—C2—N1	118.11 (11)	C17—C16—C15—C14	0.9 (2)
C13—C3—C2—N1	−117.09 (11)	C13—C14—C15—C16	0.5 (2)
C3—N2—C1—N1	0.33 (15)		

### 3-Ethyl-2-(methylsulfanyl)-5,5-diphenyl-3H-imidazol-4(5H)-one Hydrogen-bond geometry (Å, º)

**Table:**

*D*—H···*A*	*D*—H	H···*A*	*D*···*A*	*D*—H···*A*
C11—H11···O1^i^	0.95	2.49	3.434 (2)	171

Symmetry code: (i) −*x*, −*y*+1, −*z*+1.

## References

[bb1] Ait Mansour, A., Elmoutaouakil Ala Allah, A., Lgaz, H., Messali, M., Lee, H., Bazzi, L., Salghi, R., Ramli, Y. & Hammouti, B. (2025). *J. Mol. Struct.* **1321**, 139910.

[bb2] Akrad, R., Guerrab, W., Lazrak, F., Ansar, M., Taoufik, J., Mague, J. T. & Ramli, Y. (2018). *IUCrData* **3**, x180934.

[bb3] Akrad, R., Mague, J. T., Guerrab, W., Taoufik, J., Ansar, M. & Ramli, Y. (2017). *IUCrData* **2**, x170033.

[bb4] Allah, A. E. M. A., Temel, E., Guerrab, W., Nchioua, I., Mague, J. T., Talbaoui, A., Alzahrani, A. Y. A. & Ramli, Y. (2024). *J. Mol. Struct.* **1312**, 138572.

[bb5] Allen, F. H., Johnson, O., Shields, G. P., Smith, B. R. & Towler, M. (2004). *J. Appl. Cryst.* **37**, 335–338.

[bb6] AlObaid, A. A., Ala Allah, A. E. M., Dahmani, K., Aribou, Z., Kharbouch, O., Khattabi, M., Galai, M., Touhami, M. E., El-Serehy, H. A., Chaouiki, A., Chafiq, M. & Ramli, Y. (2024). *Mater.**Today Commun.* **41**, 110698.

[bb7] Asim Kaplancikli, Z., Dilek Altintop, M., Ozdemir, A., Turan-Zitouni, G. I., Khan, S. & Tabanca, N. (2012). *Lett. Drug. Des. & Discov.* **9**, 310–315.

[bb8] Brandenburg, K. & Putz, H. (2012). *DIAMOND.* Crystal Impact GbR, Bonn, Germany.

[bb9] Bruker (2016). *APEX3* and *SAINT.* Bruker AXS, Madison, Wisconsin, USA.

[bb10] El Kaouahi, S., Er-rahmany, N., Ala Allah, A. E. M., Yaqouti, S., Nounah, M., Touir, R., Larhzil, H., Saranya, J., Abuelizz, H. A., Ramli, Y., Zarrouk, A. & El Kafssaoui, E. H. (2025). *Int. J. Electrochem. Sci.* **20**, 101026.

[bb11] El Moutaouakil Ala Allah, A., Guerrab, W., Alsubari, A., Mague, J. T. & Ramli, Y. (2023). *IUCrData* **8**, x230208. 10.1107/S2414314623002080 PMC10171326 37180352

[bb12] El Moutaouakil Ala Allah, A., Guerrab, W., Maatallah, M., Mague, J. T., Talbaoui, A., Alzahrani, A. Y. A. & Ramli, Y. (2024). *J. Mol. Struct.* **1310**, 138324.

[bb13] El Moutaouakil Ala Allah, A., Kariuki, B. M., Ameziane El Hassani, I., Alsubari, A., Guerrab, W., Said, M. A. & Ramli, Y. (2024). *IUCrData* **9**, x241015. 10.1107/S2414314624010150 PMC11660168 39712662

[bb14] El Moutaouakil Ala Allah, A., Mortada, S., Tüzün, B., Guerrab, W., Qostal, M., Mague, J. T., Talbaoui, A., Yahya Abdullah Alzahrani, A., Faouzi, M. E. A. & Ramli, Y. (2025). *J. Mol. Struct.* **1335**, 141995.

[bb15] Ettahiri, W., Ala Allah, A. E. M., Lazrak, J., Safir, E.-H., Yadav, K. K., Mansour, L., Al-Tamimi, J. H., Rais, Z., Ramli, Y. & Taleb, M. (2025). *Colloids Surf. A Physicochem. Eng. Asp.* **707**, 135816.

[bb16] Farrugia, L. J. (2012). *J. Appl. Cryst.* **45**, 849–854.

[bb17] Groom, C. R., Bruno, I. J., Lightfoot, M. P. & Ward, S. C. (2016). *Acta Cryst.* B**72**, 171–179. 10.1107/S2052520616003954 PMC4822653 27048719

[bb18] Guerrab, W., Chung, I.-M., Kansiz, S., Mague, J. T., Dege, N., Taoufik, J., Salghi, R., Ali, I. H., Khan, M. I., Lgaz, H. & Ramli, Y. (2019). *J. Mol. Struct.* **1197**, 369–376.

[bb19] Guerrab, W., El Moutaouakil Ala Allah, A., Alsubari, A., Mague, J. T. & Ramli, Y. (2022). *IUCrData* **7**, x220598. 10.1107/S2414314622005983 PMC9462038 36339896

[bb20] Guerrab, W., El Moutaouakil Ala Allah, A., Alsubari, A., Mague, J. T. & Ramli, Y. (2023). *IUCrData* **8**, x230125. 10.1107/S2414314623001256 PMC9993893 36911084

[bb21] Guerrab, W., Mortada, S., El Moutaouakil Ala Allah, A., Demirtaş, G., Mague, J. T., Alzahrani, A. Y. A., AL Mughram, M. H., Faouzi, M. E. A. & Ramli, Y. (2025). *J. Mol. Struct.* **1333**, 141802.

[bb22] Karolak-Wojciechowska, J. & Kieć-Kononowicz, K. (1987). *J. Crystallogr. Spectrosc. Res.* **17**, 485–494.

[bb23] Karolak-Wojciechowska, J., Mikołajczyk, M., Zatorski, A., Kieć-Kononowicz, K. & Zejc, A. (1985). *Tetrahedron* **41**, 4593–4602.

[bb24] Kieć-Kononowicz, K., Zejc, A., MikoŁajczyk, M., Zatorski, A., Karolak-Wojciechowska, J. & Wieczorek, M. W. (1981). *Tetrahedron* **37**, 409–415.

[bb25] Krause, L., Herbst-Irmer, R., Sheldrick, G. M. & Stalke, D. (2015). *J. Appl. Cryst.* **48**, 3–10. 10.1107/S1600576714022985 PMC4453166 26089746

[bb26] Shankaraiah, N., Nekkanti, S., Chudasama, K. J., Senwar, K. R., Sharma, P., Jeengar, M. K., Naidu, V. G. M., Srinivasulu, V., Srinivasulu, G. & Kamal, A. (2014). *Bioorg. Med. Chem. Lett.* **24**, 5413–5417. 10.1016/j.bmcl.2014.10.038 25453799

[bb27] Sheldrick, G. M. (2015*a*). *Acta Cryst.* A**71**, 3–8.

[bb28] Sheldrick, G. M. (2015*b*). *Acta Cryst.* C**71**, 3–8.

[bb29] Spackman, P. R., Turner, M. J., McKinnon, J. J., Wolff, S. K., Grimwood, D. J., Jayatilaka, D. & Spackman, M. A. (2021). *J. Appl. Cryst.* **54**, 1006–1011. 10.1107/S1600576721002910 PMC8202033 34188619

[bb30] Spek, A. L. (2020). *Acta Cryst.* E**76**, 1–11. 10.1107/S2056989019016244 PMC6944088 31921444

[bb31] Tan, S. L., Jotani, M. M. & Tiekink, E. R. T. (2019). *Acta Cryst.* E**75**, 308–318. 10.1107/S2056989019001129 PMC6399703 30867939

[bb32] Westrip, S. P. (2010). *J. Appl. Cryst.* **43**, 920–925.

